# Toll-Like Receptor 2 Induced Angiogenesis and Invasion Is Mediated through the Tie2 Signalling Pathway in Rheumatoid Arthritis

**DOI:** 10.1371/journal.pone.0023540

**Published:** 2011-08-17

**Authors:** Tajvur Saber, Douglas J. Veale, Emese Balogh, Jennifer McCormick, Sinead NicAnUltaigh, Mary Connolly, Ursula Fearon

**Affiliations:** Department of Rheumatology, Dublin Academic Medical Centre and The Conway Institute of Biomolecular and Biomedical Research, University College Dublin, Dublin, Ireland; University of Michigan School of Medicine, United States of America

## Abstract

**Background:**

Angiogenesis is a critical early event in inflammatory arthritis, facilitating leukocyte migration into the synovium resulting in invasion and destruction of articular cartilage and bone. This study investigates the effect of TLR2 on angiogenesis, EC adhesion and invasion using microvascular endothelial cells and RA whole tissue synovial explants *ex-vivo*.

**Methods:**

Microvascular endothelial cells (HMVEC) and RA synovial explants *ex vivo* were cultured with the TLR2 ligand, Pam3CSK4 (1 µg/ml). Angiopoietin 2 (Ang2), Tie2 and TLR2 expression in RA synovial tissue was assessed by immunohistology. HMVEC tube formation was assessed using Matrigel matrix assays. Ang2 was measured by ELISA. ICAM-1 cell surface expression was assessed by flow cytometry. Cell migration was assessed by wound repair scratch assays. ECM invasion, MMP-2 and -9 expression were assessed using transwell invasion chambers and zymography. To examine if the angiopoietin/Tie2 signalling pathway mediates TLR2 induced EC tube formation, invasion and migration assays were performed in the presence of a specific neutralising anti-Tie2mAb (10 ug/ml) and matched IgG isotype control Ab (10 ug/ml).

**Results:**

Ang2 and Tie2 were localised to RA synovial blood vessels, and TLR2 was localised to RA synovial blood vessels, sub-lining infiltrates and the lining layer. Pam3CSK4 significantly increased angiogenenic tube formation (p<0.05), and upregulated Ang2 production in HMVEC (p<0.05) and RA synovial explants (p<0.05). Pam3CSK4 induced cell surface expression of ICAM-1, from basal level of 149±54 (MFI) to 617±103 (p<0.01). TLR-2 activation induced an 8.8±2.8 fold increase in cell invasion compared to control (p<0.05). Pam3CSK4 also induced HMVEC cell migration and induced MMP-2 and -9 from RA synovial explants. Neutralisation of the Ang2 receptor, Tie2 significantly inhibited Pam3CSK4-induced EC tube formation and invasion (p<0.05).

**Conclusion:**

TLR2 activation promotes angiogenesis, cell adhesion and invasion, effects that are in part mediated through the Tie2 signalling pathway, key mechanisms involved in the pathogenesis of RA.

## Introduction

Rheumatoid Arthritis (RA) is a chronic progressive autoimmune disease characterised by proliferation of synovial membrane (SM), which leads to degradation of articular cartilage and subchondral bone. Angiogenesis is an early event required for pannus development in RA enabling activated monocytes to enter the SM *via* endothelial cells (EC) by active recruitment [1], [2]. RA synoviocytes manifest an abnormal phenotype characterised by increased proliferation, resistance to apoptosis and invasiveness of adjacent tissue. This leads to a self perpetuating and persistent infiltration of immune cells resulting in synoviocyte hyperplasia which transforms SM into an aggressive, tumour-like tissue - ‘pannus’ which is capable of destroying adjacent articular cartilage and bone. Pro-inflammatory cytokines, such as TNF-α and IL-1β, are key mediators of these processes, however, it remains unclear which mechanisms are involved in the initiation and regulation of cytokine production and other tissue-destructive mediators [3]–[5].

Toll-Like receptors (TLRs) have been implicated in the pathogenesis of RA with studies showing increased TLR2 and TLR4 expression in the perivascular regions of the joint [6], at the sites of attachment and invasion into cartilage/bone, and on synovial macrophages [7]. TLR2 ligand bacterial peptidoglycan (PG) has been detected in RA synovial fluids [8]. Increased expression of TLR2 has been demonstrated in collagen induced arthritis, and TLR2 deficient mice do not develop streptococcal cell wall (SCW) induced arthritis [9]. Furthermore, it has been shown that dominant negative forms of the essential TLR2 adapter molecules MyD88 and MAL/TIRAP ablate pro-inflammatory cytokine production in RA synoviocytes demonstrating that TLR2 and TLR4 signalling pathways are functional in these cells [10].

Angiogenesis is one of the earliest events in the initiation of synovial inflammation [1]. Several studies have demonstrated that angiogenesis is highly dysregulated in RA, which is associated with increased differential expression of pro-angiogenic factors in synovial fluid and tissue such as VEGF, Angiopoietin 1/2, PlGF, PDGF or TGFβ1, that can promote either immature or mature stable vessels [11]–[18]. More recently studies have suggested that synovial angiogenesis maybe regulated by TLR2 activation. TLR2 is expressed in SM perivascular regions [19], and *in vitro* studies confirm that TLR2 activation induces VEGF/IL-8 expression in synovial fibroblasts and chondrocytes [20], [21], and MMP-9 in corneal epithelial cells and THP-1 macrophages [22]. The aim of this study is to examine the effects of TLR2 activation on angiogenic processes using RA synovial explants and HMVEC cultures. We demonstrate using RA whole tissue SM explants that TLR2 activation induces Ang2 and MMP2, 9 expression, critical factors involved in blood vessel destabilisation and advancement through the inflamed SM. Furthermore we demonstrate that TLR2 activation significantly induces angiogenic tube formation, Ang2, ICAM-1 cell surface expression, EC invasion and migration, effects that in part inhibited by Tie2 receptor blockade. These results suggest that TLR2 induced angiogenic processes are in part mediated through the Tie2 signalling pathway.

## Materials and Methods

### Arthroscopy and sample collection

RA patients with clinically active inflamed knees prior to biologic therapy were recruited from rheumatology outpatient clinics at St. Vincent's University Hospital. Following approval by the institutional ethics committee, all patients gave written informed consent. All treatment was fully compliant with the Helsinki Declaration. RA synovial biopsies were obtained at arthroscopy as previously described and were either placed in cryopreservation embedding media OCT compound (Tissue Tek, The Netherlands) for histological analysis or established as RA whole tissue synovial explants cultures established [23].

### Immunohistochemistry

7 µm OCT sections were placed on glass slides coated with 2% 3-amino-propyl-triethoxy-silane (Sigma-Aldrich, St. Louis, MO) and dried overnight at room temperature. Sections were stored at −80°C until required for staining. Tissue sections were allowed to reach room temperature, fixed in acetone for 10 minutes and air-dried. Non-specific binding and endogenous peroxidase activity was blocked using 10% casein and 0.3% H_2_O_2_ respectively. A routine three-stage immunoperoxidase labelling technique incorporating avidin-biotin-immunoperoxidase complex (DAKO, Glostrup, Denmark) was used. The sections were incubated with primary antibodies against mouse-monoclonal Ang2 (R&D systems, UK), mouse-monoclonal Tie2 (R&D systems, UK) and mouse monoclonal anti-TLR2 antibody (kind gift from OPSONA therapeutics, Ireland) at room temperature for 1 hour. Sections were also incubated with an appropriate isotype matched mouse monoclonal antibody as a negative control. Colour was developed in solution containing diaminobenzadine-tetrahydrochloride (Sigma-Aldrich, St. Louis, MO), 0.5% H_2_O_2_ in PBS buffer (pH7.6). Slides were counterstained with haematoxylin and mounted.

### RA ST Explant Culture

To examine the effect of Pam3CSK4 on Angiopoietin 2, MMP-2 and MMP-9 expression in RA synovial tissue we established an *ex vivo* RA synovial tissue explant model, which maintains the synovial architecture and cell-cell contact, and therefore more closely reflects the *in vivo* environment [23]. RA synovial explant tissue was sectioned into 1 mm cubes in 96 well plates (Falcon, Franklin Lakes, NJ) in RPMI 1640 supplemented with streptomycin (100 units/ml) and penicillin (100 units/ml) and incubated with Pam3CSK4 (1 µg/ml) for 24 hours at 37°C in 5% CO2. Previous studies using RA synovial explants cultured with Pam3CSK4 (0.2 ug/ml–10 ug/ml) demonstrated maximal pro-inflammatory effects at a concentration of 1 ug/ml [24]. Supernatants were harvested and assayed for Ang2 by ELISA and MMP2 and 9 by zymography.

### Isolation and Culture of HMVEC

Human microvascular endothelial cells HMVEC (Cat.No CC-2543) (Clonetics, Lonza, Waterville, Inc), were grown in endothelial basal medium (EBM) supplemented with 5% FCS, 0.5 ml human epidermal growth factor (hEGF), 0.5 ml hydrocortisone, 0.5 ml gentamicin, 0.5 ml bovine brain extract (Clonetics, Lonza, Waterville, Inc) and were used for experiments between the passages 3–8.

### Matrigel *in vitro* HMVEC tube formation assay

Matrigel (Becton Dickenson) basement membrane matrix was used to examine HMVEC tube formation in response to Pam3CSK4. Matrigel (50 µl) was plated in 96 well culture plates slides and allowed to polymerise at 37°C in 5% CO_2_ humidified for 30 mins. HMVEC were removed from culture, trypsinised, and resuspended at 4×10^4^ cells/ml in EBM medium containing 2% FCS (Clonetics). Four hundred microlitres of cell suspension was added to each chamber, followed by addition of Pam3CSK4 (1 µg/ml) and incubated for 24 h at 37°C in 5% CO_2_. Endothelial cell tube formation was examined using phase contrast microscopy and photographed. A connecting branch between two discrete EC was counted as one tube and required a consistent intensity and thickness as previously described [23]. The tube analysis was determined from 5 sequential high powered fields (Magnification×10) focusing on the surface of the matrigel. To assess the effect of TLR2 blockade cells were incubated with. To assess if the angiopoietin/Tie2 signalling pathway mediates Pam3CSK4 induced angiogenesis, experiments were also performed in the presence of a specific mouse anti-Tie2 mAb (10 ug/ml) and a isotype matched IgG control mAb (10 ug/ml).

### Angiopoeitin2 ELISA

Angiopoietin2 was measured by specific Quantikine ELISA as per manufacturer's instructions (R&D systems UK). ELISA standards ranged from 78–5000 pg/ml. The absorbance was measured at 450 nm.

### Flow cytometric analysis of ICAM-1 on HDEC

HMVEC were plated to a cell count of 5×10^4^ in 12 well plates (Falcon), and allowed to grow to confluence. HMVEC were incubated in serum reduced EBM for 24 hours (1%FCS) and then incubated for a further 24 hrs with Pam3CSK4 (1 µg/ml). Cells were then harvested with a cell scraper and transferred to fluorescence-activated cell sorting tubes (Becton Dickinson). Cells were then washed and incubated with an optimal concentration of phycoerythrin-conjugated monoclonal mouse anti-ICAM-1 and isotype-matched control (Becton Dickinson) for 30 mins at 4°C. Samples were then washed twice with 1% PBA, and fixed in 1% PFA. ICAM cell surface expression was quantified using a FACScan flow cytometer (Becton Dickinson) using Lysis II software. Further experiments examined the effect of Pam3CSK4 induced ICAM-1, in the presence of anti-Tie2 mAb (10 ug/ml) or a isotype matched IgG control mAb (10 ug/ml).

### Transwell Invasion Assay

Biocoat Matrigel Invasion Chambers (Becton Dickinson, UK) were used to assess endothelial cell migration in response to Pam3CSK4. Cells were seeded at a density of 2.5×10^4^ per well in the migration chamber on 8 µm membranes pre-coated with matrigel. EGM containing Pam3CSK4 (1 µg/ml) was placed in the lower well of the chamber. Cells were allowed to migrate for 24 hours in EBM medium containing 1% FCS. Non-migrating HMVECs were removed from the upper surface by gentle scrubbing. Migrating cells attached to the lower membrane were fixed with 1% glutaraldehyde and stained with 0.1% crystal violet. To assess the average number of migrating cells, cells were counted in five random high power fields (hpf). To examine if the angiopoeitin/Tie2 signalling pathway mediates Pam3CSK4 induced cell invasion, experiments were performed in the presence of a anti-Tie2 (10 ug/ml) neutralising mAb or isotype matched IgG control mAb (10 ug/ml)

### Wound repair assays

HMVEC were seeded onto 48 well plates and allowed to come to confluence. A single scratch wound was induced through the middle of each well with a sterile pipette tip. Cells were subsequently stimulated for 24 hrs with Pam3CSK (1 µg/ml). HMVEC migration across the wound margins from 12–24 hrs was assessed and photographed.

### Zymography

Cultured supernatants from RA synovial explants incubated with Pam3CSK4 were separated by electrophoresis under nonreducing conditions by SDS-PAGE in 10% polyacrlyamide gels copolymerised with 1% gelatine. Gels were vigorously washed twice for 25 minutes in 2.5% Triton X-100 to remove SDS, rinsed for 25 minutes in dH_2_O, then incubated overnight in 50 mM Tris, 50 mM NaCl, 10 mM CaCl_2_, pH 7.5 at 37°C. Following incubation gels were rinsed for 5 minutes in dH_2_O before addition of Coomassie blue stain (30% Isopropanol, 10% Acetic Acid, 0.25 mg/ml Brilliant Blue R) for 10 mins. Gels were visualised using the UVP Bioimaging AutoChemi system (UVP, Cambridge, UK).

### Statistical Analysis

SPSS15 system for windows was used for statistical analysis. Non-parametric Wilcoxon Signed Rank test for related samples was used for analysis of RA synovial explant cultures. Parametric student *t*-tests were used for analysis of HMVEC data. *P* values less than 0.05 were considered significant.

## Results

### Pam3CSK4 induces *in vitro* angiogenesis

**Figure 1:**
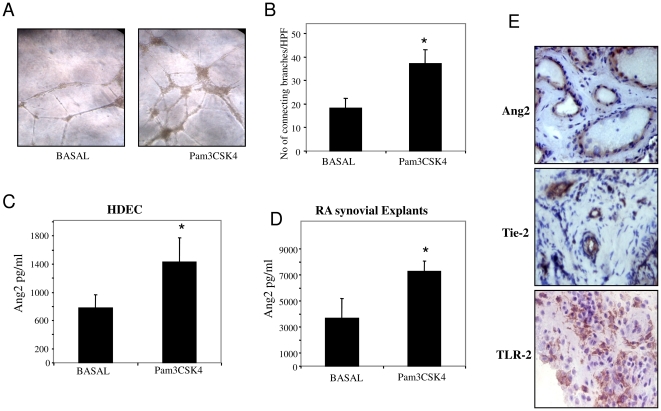
TLR2 activation induces EC tube formation and Ang2 expression. Human dermal microvascular endothelial cell tubule formation on matrigel matrix following stimulation with Pam3CSK4 (1 ug/ml). (A) Representative image of baseline tube formation (left panel) and tube formation following stimulation with Pam3CSK4 (right panel). (B) Quantitative analysis of the number of connecting branches at baseline and in response to Pam3CSK4. The tube analysis was determined from 5 sequential fields (Magnification×40) focussing on the surface of the matrigel (n = 4). (C–D) The effect of Pam3CSK4 on angiopoietin-2 expression in HMVEC (n = 4) and RA synovial explants (n = 6). Data represented as the mean+/−sem. *p<0.05 significantly different from baseline. (E) Ang2, Tie2 and TLR2 expression in RA synovial tissue sections.

Induction of HMVEC tube formation on Matrigel matrices plated in 96-well culture plates was assessed following stimulation with Pam3CYSK4 (1 ug/ml). Tube like structures formed after 24 hrs incubation. Figure 1A shows representative images of increased tube formation following Pam3CSK4 stimulation as demonstrated by an increase in the number of connecting branch between two endothelial cells. Figure 1B graphically illustrates tube formation quantification, demonstrating significant induction in response to Pam3CSK4 (p<0.05). To determine if Pam3CSK4 directly induces growth factor secretion in inflammatory cells, the expression of Ang2 in response to stimulation by Pam3CSK4 in HMVEC and in the *ex-vivo* RA synovial explants cultures was assessed. Pam3CSK4 significantly increased Ang2 expression in HMVEC (Figure 1C) and in the *ex-vivo* RA synovial explants (Figure 1D) (all p<0.05). To demonstrate expression of Ang2, its receptor Tie2 and TLR2 in RA synovial tissue sections, immunohistochemical analysis was performed. Ang2 and Tie2 were localised to the blood vessels and to a lesser extent the lining layer. Figure 1E shows representative images of strong blood vessel expression for Ang2 and Tie2 in RA synovial tissue sections. TLR2 expression was localised to the blood vessels, sub-lining infiltrates and to the lining layer (Figure 1E).

### Pam3CSK4 induces ICAM-1 on HDEC

**Figure 2:**
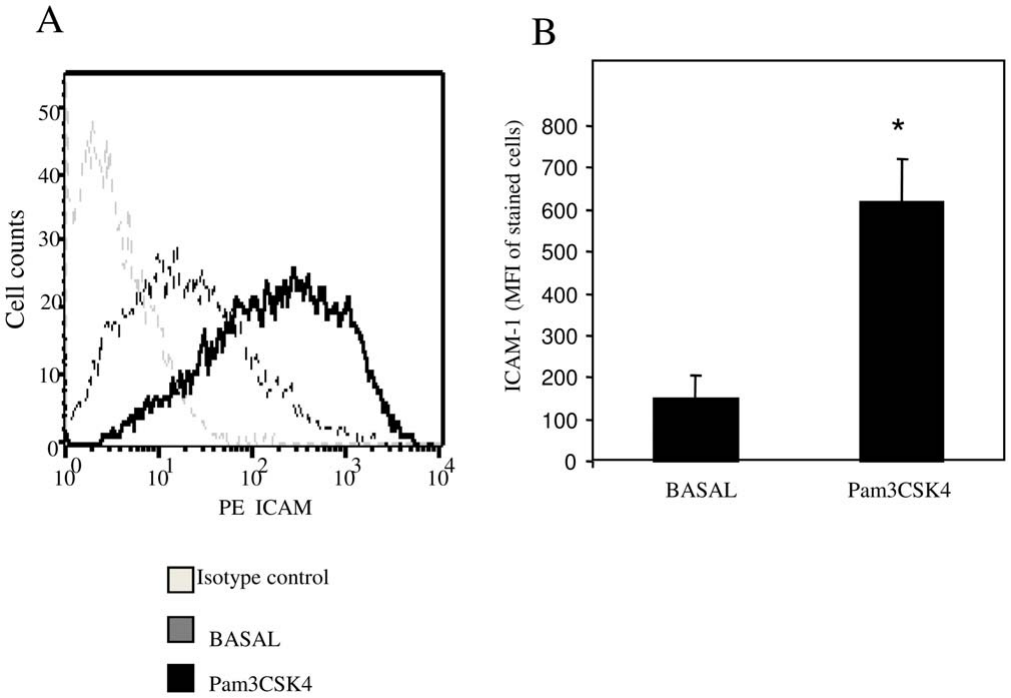
TLR2 activation induces ICAM-1 cell surface expression HMVEC. Human dermal microvascular endothelial cells were stimulated with Pam3CSK4 (1 ug/ml) and expression of ICAM-1 was detected by flow cytometric analysis. (A) Representative flow cytometry histogram demonstrating induced ICAM-1 expression on HMVEC following stimulation with Pam3CSK4 (black line) compared to basal (grey line). **B**. Quantification of ICAM-1 expression following incubation with Pam3CSK4. Data represented as mean fluorescent intensity (mean±sem, n = 4). * p<0.05 significantly different from unstimulated.

To assess whether Pam3CSK4 can induce adhesion molecules in HMVEC, we assessed ICAM-1 cell surface expression using FACS analysis. Figure 2A shows a representative histogram demonstrating increased cell surface expression of ICAM in response to Pam3CSK4 compared to basal or control IgG. Pam3CSK4 stimulation significantly increased ICAM-1 expression from an MFI intensity of 149±54 to 617±103 cells, (Fig. 2A) (p<0.05) following 24 hrs stimulation.

### Pam3CSK4 induces EC invasion and migration

**Figure 3:**
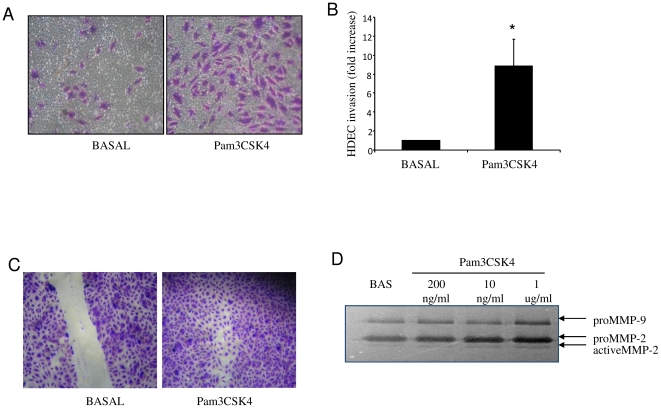
TLR-2 activation induces EC invasion and migration and MMP-2 and 9 expression in RA synovial explants. (**A**) Representative photomicrograph shows HMVEC invasion following Pam3CSK4 (1 µg/ml), stimulation. At 24 hours invading cells attached to lower membrane were fixed (1% glutaraldehyde) and stained (0.1% crystal violet) (Mag×40). (**B**) Representative bar graph quantifying HMVEC invasion. (n = 4). *p<0.05 significantly different to control. (C) Representative photomicrograph showing cells repopulating the wound in response to Pam3CSK4 (1 ug/ml). (D) Representative gel of MMP-2 and 9 activity by gelatine zymography in response to Pam3CSK4 in RA synovial explants (n = 3).

Angiogenesis and EC activation are essential steps in the progression of RA, therefore to further assess the potential role of TLR-2 activation in inducing these processes, the effect of Pam3CSK4 on HMVEC cell invasion and migration was assessed using transwell matrigel™ invasion chambers, wound repair assays and zymography. HMVEC were incubated with Pam3CYSK (1 ug/ml) for 24 hrs where cells migrated through a 8 µm membranes pre-coated with matrigel. Following incubation, cells were fixed and stained with crystal violet. Figure 3A shows representative images of increased HMVEC invasion under basal condition where a minimal number of stained EC were observed compared to Pam3CSK4 stimulated cells where a significant increase in the number of invading ECs is demonstrated. HMVEC invasion quantification was significantly induced by Pam3CSK4 (1 µg/ml) where cell invasion increased 8.8±2.8 fold compared to control (Figure 3B). To examine the effect of Pam3CYSK4 on HMVEC migration wound repair assays were performed. A wound was created through the middle of each well, and cells were cultured with Pam3CYSK4 from 12–24 hrs. Migration across the wound margin and repopulation of ECs was assessed. Figure 3C shows a clear wound under basal condition, where minimal migration of cells across the wound margin was observed. In contrast Pam3CSK4 induced cell migration across the wound margins resulting in almost complete closure of the wound. Finally using gelatin zymography we examined if Pam3CSK4 (200 ng/ml–1 ug/ml) induced MMP-2 and -9 expression, which are key metalloproteinases involved in angiogenesis and blood vessel invasion through the extracellular matrix. Figure 3D shows representative zymography gel images demonstrating that Pam3CSK4 at 1 ug/ml induces both MMP-2 and -9 in *ex vivo*- RA synovial explants. While both pro-MMP-2 and -9 forms were induced, only active MMP-2 was induced by Pam3CSK4.

**Figure 4:**
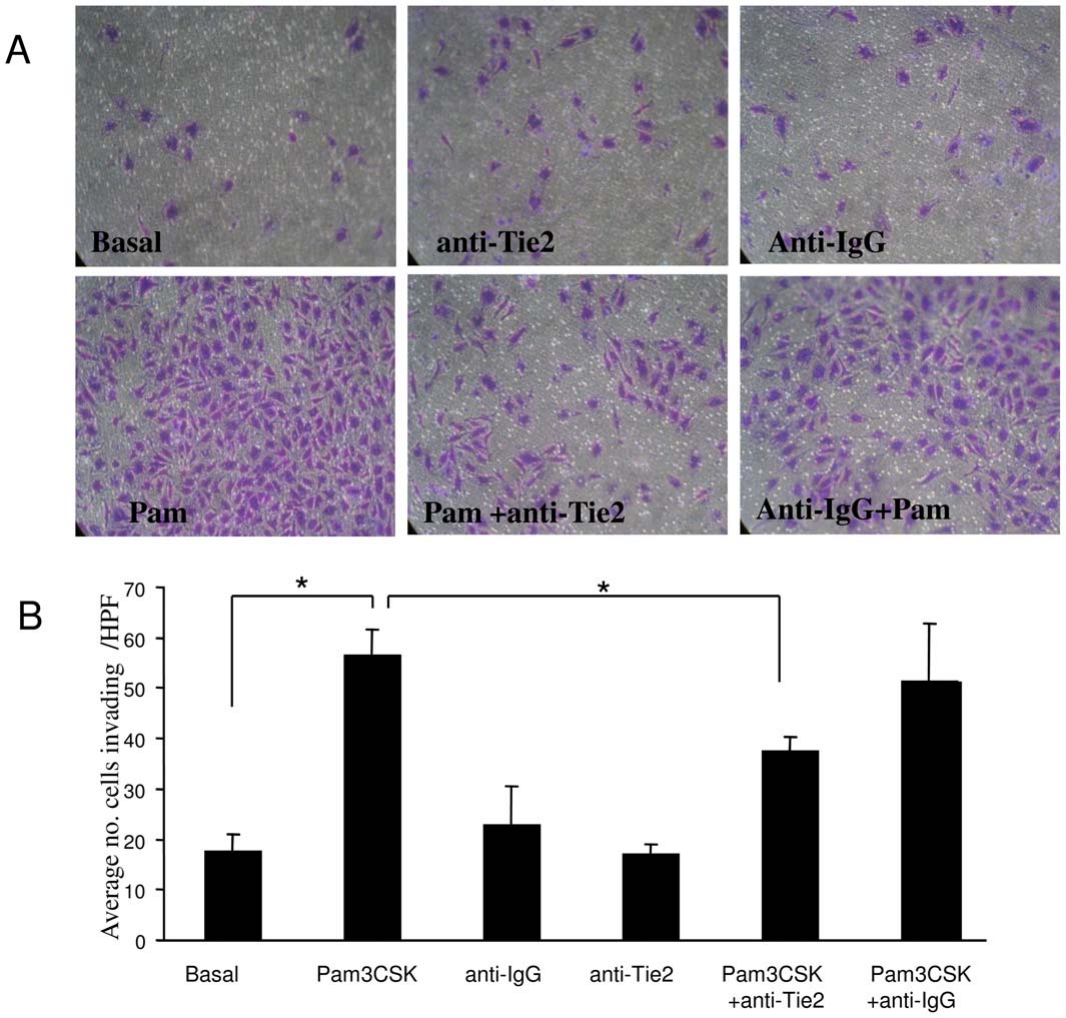
TLR2 induced EC invasion is inhibited by anti-Tie2. (A) Representative photomicrographs showing anti-Tie2 blocks Pam3CSK4 induced HMVEC invasion, with no effect observed for IgG control mAb. At 24 hours invading cells attached to lower membrane were fixed (1% glutaraldehyde) and stained (0.1% crystal violet) (Mag×40). (B) Representative bar graph quantifying HMVEC invasion. (n = 4). *p<0.05 significantly different.

**Figure 5:**
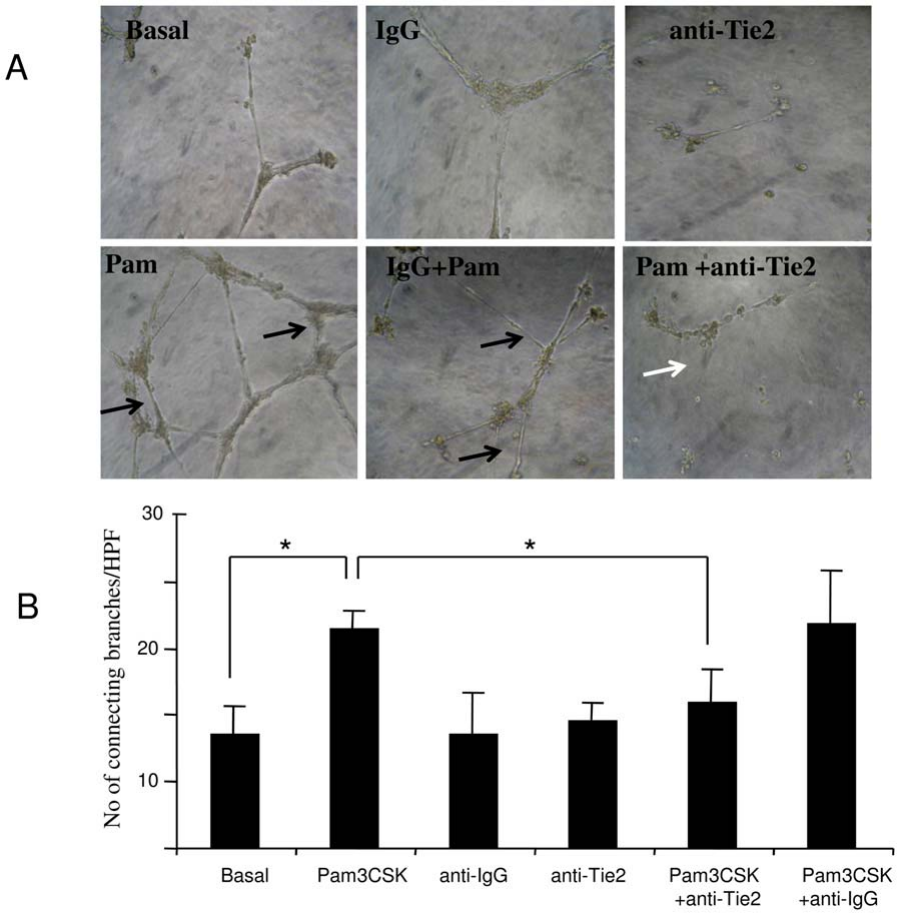
TLR2 induced EC tube formation and wound repair are blocked by anti-Tie2. (**A**) Representative photomicrographs showing anti-Tie2 blockade of TLR-2 induced angiogenesis, with effect observed for IgG control mAb (n = 3 experiments). Black arrows indicates an increase in EC-EC connecting branches and tube formation in response to PAM3CSK4, white arrow indicates a decrease in the number of connecting branches in the presence of an-Tie2. (B) Representative bar graph quantifying HMVEC tube formation *p<0.05 significantly different.

Finally, to examine if the Ang2/Tie2 pathway mediates TLR2 induced angiogenesis and invasion, HMVEC were cultured with Pam3CSK4 in the presence or absence a specific anti-Tie2 antibody. Minimal invasion was observed under basal condition or when incubated with Anti-Tie2 or Anti-IgG alone. However we demonstrated that Pam3CSK4- significantly induced EC invasion an effect that was inhibited in the presence of anti-Tie2 (p<0.05) with no effect observed with the isotype matched IgG control (Fig. 4). Furthermore Pam3CSK4-induced tube formation (black arrows) was inhibited in the presence of anti-Tie2 (white arrows) with no effect observed for the isotype matched control (Fig. 5). Anti-Tie2 had no effect on Pam3CSK4 induced ICAM-1 expression (data not shown). This data confirms that Pam3CSK4 induced EC function is in part mediated by the Ang2/Tie2 signalling pathway.

## Discussion

Angiogenesis, cell adhesion and migrational processes play a critical role in the pathogenesis of RA. TLR2 has been implicated in the pathogenesis of RA, yet a role for TLR2 in angiogenesis and EC activation has not been extensively studied. In this study we demonstrate that TLR2 induces angiogenic tube formation and Angiopoietin 2 expression which is a key growth factor involved in new blood vessel formation and destabilisation. We demonstrate increased ICAM-1 expression which is involved in leukocyte migration in the inflamed joint. We demonstrate that TLR2 activation induces EC invasion and migration, in addition to increased MMP-2 and -9 expression by RA synovial explants. Finally, we show that TLR2-induced angiogenesis and invasion is blocked by the presence of a specific anti-Tie2 monoclonal antibody.

The identification of pattern recognition TLRs in the RA joint suggests they may play an important role in mediating inflammatory responses in RA. Several studies have shown TLR2 expression in RA synovial tissue and have shown that TLR2 activation in RA synovial explants, RASFCs and macrophages induces the MyD88/MAL pathway [6]–[10], [19], [24]. In animal models intra-articular injection of the TLR2 leads to development of destructive arthritis in mice and TLR2/MyD88 knockout mice are protected from SWC induced joint inflammation [8]. Several potential TLR2 ligands have been implicated, including Acute Serum Amyloid-A, Heat Shock protein/GP96, Fibronectin fragments, Hyalauronidase oligosaccharides and HMBG-1, all of which are highly expressed in RA synovial fluid [25]–[29]. The existence of a ligand is further supported by studies which show that conditioned media from RA synovial explants can activate macrophages in a MyD88 and Mal dependent manner [6].

Angiogenesis is one of the earliest events in RA, facilitating leukocyte extravasation, synovial proliferation and pannus formation [1]–[5]. Recruitment of inflammatory cells into the joint is mediated by adhesion molecule/ligand pairs and chemokines [30]. Previous studies have demonstrated that TLR2 can induce one of the main angiogenic growth factors VEGF, and the chemokine IL-8, in fibroblast, chondrocytes, in corneal epithelial cells and THP-1 macrophages [21], [22], [31]. Consistent with these studies a recent report by Grote et al, demonstrated that the TLR2/6 agonist macrophage-activating lipopeptide of 2 kDa (MALP-2), induced angiogenic tube formation and leukocytes migration *in vivo* and *in vitro* an effect mediated through GM-CSF pathways [32]. In this study we demonstrate that TLR2 activation induces EC tube formation and a key angiogenic growth factor Ang2. A close relationship exists between the differential vascular morphology within the inflammatory joint and the differential expression of angiogenic growth factors such as VEGF, angiopoietins; Ang-1 and Ang-2, FGF-1, TGFβ-1 and PDGF-1 [1], [11]–[13], [24], [33]. Through its receptor Tie-2, Ang2 acts in the presence of abundant VEGF on invading vascular sprouts by blocking the maturation and stabilisation process, [15], [16], these vessels remain in a ‘plastic’ state, in which they may become more responsive to a sprouting signal provided by VEGF. Ang2 can also enhance chemotactic migration of RASFC and can trigger EC permeability and recent studies have demonstrated that in addition to its pro-angiogenic properties, Ang2 can sensitise cells to TNFα stimulation, suggesting it plays an important role in RA [34].

We demonstrated TLR2 activation-induced ICAM-1 expression on EC suggesting a potential mechanism whereby TLR2 promotes cell migration. This is consistent with studies using mouse and human lung ECs where they demonstrate that TLR2 activation results in ICAM-1, VCAM-1 and chemokine expression, resulting in neutrophil trans-endothelial and leukocyte recruitment [35], [36]. In atherosclerosis models, TLR2 activation of monocytes resulted in an increase in adhesive and migratory capacity of cells [37]. Furthermore in primary synovial fibroblasts PG activation induced ICAM-1, IL-6 and IL-8 [38]. These results along with TLR2 induction of EC tube formation and Angiopoietin 2 suggest that angiogenesis and leukocyte recruitment to the joint may in part be mediated by TLR2.

Furthermore, we demonstrate that Pam3CSK4 significantly induces cell invasion, cell migration and MMP-2 and -9 secreted expression. Cell motility and ECM degradation are essential for EC migration during angiogenesis and for the advancement of the newly formed vessels through the synovial tissue [25], [39]–[41]. The invasion of blood vessels through the inflamed SM is facilitated by induced expression of gelatin type matrix metalloproteinases MMP-2 and -9. *In vivo* and *in vitro* studies have highlighted the role of MMP-2 and 9 in angiogenesis and in the pathogenesis of RA. MMP-2 and -9 are highly expressed in RA SM atherosclerotic plaques and are upregulated during vascular injury [41]–[44], Blockade of TLR2 signalling via MyD88, results in inhibition of MMP-2 and -9 in a culture model of atherosclerosis [32], thus further supporting the hypothesis that TLR2 blockade may represent a potential therapeutic strategy for atherosclerosis, autoimmune diseases and other vascular diseases.

Finally we demonstrated that TLR2 induced angiogenesis and invasion were mediated through the Tie2 signalling pathway. The effect of Tie2 blockade on TLR2 induced angiogenic mechanisms, is most likely through TLR2 regulation of the Tie2 ligand Ang2, as demonstrated in the RA synovial explant and endothelial cell cultures. It is well established that modulation of the Tie2 receptor by its ligand Ang2 is crucial for regulation of angiogenesis, blood vessel maturation and vascular integrity [15], [45]–[47], however this is the first report to demonstrate that it mediates TLR2 downstream function. Several studies have demonstrated the presence of Tie2 and its ligands in synovial tissue, synovial fibroblast and RA osteoblasts [12], [48], [49]. *In vitro and in vivo* studies, have shown TNFα induces Tie2, Ang1 and Ang2 expression in RA synovial fibroblast [50]–[53]. Blocking Tie2 activation in a collagen induced arthritis model inhibits arthritis-induced angiogenesis, RANKL expression leading to a reduction in bone erosion suggesting Tie2 as a potential therapeutic target [52], [53]. Ang2 deficient mice have an impaired ability to express cytokine-inducible adhesion molecules [34]. Furthermore, Ang2 can also sensitize cells to the actions of TNFα, where Ang2 promoted TNFα-induced leukocyte rolling and adhesion molecule expression [34]. These findings identify Tie2 as a key signalling pathway in inflammatory arthritis and imply that interventions designed to target the upstream triggers of the Tie2 pathway could be clinically beneficial.

In conclusion we have demonstrated that TLR-2 activation plays a key role in several important mechanisms involved in angiogenesis and advancement of blood vessel through the inflamed synovial tissue effects that may in part be mediated by the Ang2/Tie-2 signalling pathway. We have also shown the relevance of TLR2 in both microvascular endothelial cells and RA synovial cells *in vitro* and *ex vivo*. These findings provide evidence of a common molecular link between TLR2 and the pathogenesis of both vascular and joint inflammation and further highlight the therapeutic potential of targeting TLR2 in the treatment of RA.
